# Chicago Medical Society

**Published:** 1870-09

**Authors:** 


					﻿ARTICLE XXIX.
TRAUMATIC, IRIDEREMIA, AND APHAKIA.
By C. HIXSON, M.D., formerly Prof, of Ophthamology in the Kansas City
College of Physicians and Surgeons.
The infrequency of cases like the following is a sufficient
apology for its publication. Mr. B., of Kansas, is a sawyer by
occupation, and sometime in January last, was struck by a
small chip hurled with great velocity from the saw, which entered
the centre of the right eye, making an incision of more than
three-fourths of the diameter of the cornea. Being remote
from any professional assistance, his wife did the surgery in
the case. Cold applications were made at intervals for several
days, when what he describes as strings of pus was found in the
wound, and which his wife pulled away with considerable pain
on the part of the patient. The eye gradually improved, and
to-day I find the following interesting condition of affairs:
There is, extending in a vertical line, embracing about three-
fourths of the diameter of the cornea, a firm, opaque cicatrix.
There is not a vestige of the iris to be seen in the injured eye.
The iris of the left eye is a very light grey, and contrasted with
the deep, dark, blackness of the injured eye, is remarkable.
The only thing seen by lateral illumination or by the Ophthal-
moscope is a few opaque filaments of the capsule. There is
complete aphakia. Without a glass, the patient, has only quan-
tative perception of light — everything is blurred and indistinct.
With a cataract glass and through a stenopaic hole, he can
read No. 4 Sneller at 18 inches. He is very much annoyed
by the inability to focus the rays of light equally in the two
eyes, and I hope by a stenopaic catacact glass to be able to do
away with this difficultuy to a considerable extent, if not
entirely.
r or 11 a t it a s at	r 1t11t s
CHICAGO MEDICAL SOCIETY.
August 1st, 1870.—Dr. Fred. Hotz exhibited a specimen of
urinary calculus, of very unusual size, removed by him in May
last. The stone was oval in shape, perfectly smooth, 4 inches
long by 2} in breadth, about 2 inches in thickness, and weighed
12| ounces. A section through the center showed the calculus
to be composed of several distinct layers of an almost pure
white material, mostly the phosphates. A small cavity in the
center was nearly filled by an oval tuberculated nucleus, about
an inch in diameter.
Tlie patient, from whom the calculus was removed, was sixty
years of age, and in rather feeble health. Symptoms indicating
the existence of calculi had existed for many years.
When first called to see the case in April last, the Doctor
found him suffering from acute pain in the bladder, difficulty in
passing water, etc. It was found impossible to pass a catheter
or sound. At the earnest solicitation of the patient an opera-
tion was undertaken, although but little encouragement of a
favorable result could be given.
The ordinary lateral operation was begun, but on cutting into
the bladder, it was found impossible to withdraw the calculus
through the opening. An attempt was then made to crush the
stone, but with all the force that could be applied, even to the >
flattening of the teeth of the forceps, they were unable to frac-
ture it. The operation was then converted into the bilateral,
and, after considerable manipulation and some injury to the tis-
sue, the calculus was finally extracted.
The patient died two days after the operation. The autopsy
revealed considerable thickening of the walls of the bladder
and constriction of its passage from the chronic inflammation.
Both ureters and the pelves of the kidneys were considerably
dilated. There was also a considerable abscess in one kidney.
Dr. Miller exhibited a portion of the skull of a woman, sup-
posed to be a German emigrant, who was found lying on the
track of the Northwestern Railway, a short distance from the
city limits, having been run over by a train. From certain
cuts on the head, it was suspected that she had been murdered
and the body thrown across the rails to be mangled by the pass-
ing trains, in order to hide the crime. The post mortem exam-
ination revealed several clean deep cuts in the base of the skull,
passing through into the brain, narrower at their base than at
their entrance, and appearing as if made with a hatchet or
some similar instrument. It was impossible that such injuries
could have been made by the cars. The jury in the case re-
turned a verdict of death at the hands of some person or per-
sons unknown. The body was never identified, nor any clue to
the murderer(s) obtained. In answer to a query raised, the
Doctor stated that it was impossible that the cuts could have
been made after death, as in that case the edges of the wounds
would not have been everted.
Dr. M. also presented a specimen of aortic aneurism, near the
heart, showing the thickened pericardium and the rent in the
aneurismal sac, which was the cause of death.
Dr. R. G. Bogue exhibited a specimen of fluid taken from an
hepatic cyst. It was of a d-ark red color, and contained con-
siderable sediment. The fluid had not been examined chemi-
cally or microscopically.
Dr. T. D. Fitch related a case of cancer of the omentum
and other abdominal organs, in a middle-aged woman. A
tumor began in the left iliac region, which rapidly increased
until it had reached nearly across the abdomen, and had caused
an enormous distention of this cavity. The pressure of the
tumor caused much disturbance of the functions of the body,
especially pain in the stomach, difficulty in digestion, frequent
vomiting, etc. It was regarded as not improbable that it might
be an ovarian tumor, but it was finally diagnosed as malignant,
although there could not be traced any cancerous disease in
the family. The patient died five months from the beginning of
the diseased At the post mortem examination, half a gallon of
thick, red fluid escaped from the peritoneal cavity. The omen-
turn, the liver, and nearly all the abdominal organs, were infil-
trated with cancerous matter. The omentum was nearly
destroyed—“ eaten away.” The uterus was antiflexed at an
acute angle, a fact which accounts for the impossibility of pass-
ing a sound into that organ during life. Nearly the whole of
the peritonaeum was thickly studded with little tumors, which,
although never examined with the microscope, were undoubt-
edly of a malignant nature. The cancerous matter was nowhere
circumscribed, but consisted of a diffuse involement of all the
abdominal organs.
				

